# Long-term *in vivo* survival of 3D-bioprinted human lipoaspirate-derived adipose tissue: proteomic signature and cellular content

**DOI:** 10.1080/21623945.2021.2014179

**Published:** 2021-12-27

**Authors:** Karin Säljö, Peter Apelgren, Linnea Stridh Orrhult, Susann Li, Matteo Amoroso, Paul Gatenholm, Lars Kölby

**Affiliations:** a Department of Plastic Surgery, Institute of Clinical Sciences, Sahlgrenska Academy, University of Gothenburg, Gothenburg, Sweden; b Department of Plastic Surgery, Region Västra Götaland, Sahlgrenska University Hospital, Gothenburg, Sweden; c 3D Bioprinting Centre, Department of Chemistry and Chemical Engineering, Chalmers University of Technology, Gothenburg, Sweden; d Department of Clinical Chemistry and Transfusion Medicine, Institute of Biomedicine, Sahlgrenska University Hospital, Göteborg, Sweden

**Keywords:** Lipoaspirate-derived adipose tissue, 3D bioprinting, proteomics, flow cytometry, adipose-derived stem cells/ASCs, endothelial progenitor cells/EPCs

## Abstract

Three-dimensional (3D)-bioprinted lipoaspirate-derived adipose tissue (LAT) is a potential alternative to lipo-injection for correcting soft-tissue defects. This study investigated the long-term *in vivo* survival of 3D-bioprinted LAT and its proteomic signature and cellular composition. We performed proteomic and multicolour flow cytometric analyses on the lipoaspirate and 3D-bioprinted LAT constructs were transplanted into nude mice, followed by explantation after up to 150 days. LAT contained adipose-tissue-derived stem cells (ASCs), pericytes, endothelial progenitor cells (EPCs) and endothelial cells. Proteomic analysis identified 6,067 proteins, including pericyte markers, adipokines, ASC secretome proteins, proangiogenic proteins and proteins involved in adipocyte differentiation and developmental morphogenic signalling, as well as proteins not previously described in human subcutaneous fat. 3D-bioprinted LAT survived for 150 days *in vivo* with preservation of the construct shape and size. Furthermore, we identified human blood vessels after 30 and 150 days *in vivo*, indicating angiogenesis from capillaries. These results showed that LAT has a favourable proteomic signature, contains ASCs, EPCs and blood vessels that survive 3D bioprinting and can potentially facilitate angiogenesis and successful autologous fat grafting in soft-tissue reconstruction.

## Introduction

In plastic surgery, autologous fat grafting with the Coleman technique [1] is commonly used to correct soft-tissue defects [2]; however, the procedure is compromised by a high rate of graft resorption and nutritional supply challenges associated with the increased graft size [2]. The composition of cells in the grafts, presence of adipose tissue-derived stem cells (ASCs) and bioactive factors with trophic effects can influence vascularization, as well as graft retention and survival [2]. The three-dimensional (3D)-bioprinting technique can potentially contribute to the field of soft tissue reconstruction by enabling tailor-made 3D architectures of grafts that address various clinical needs with regard to the size and geometry, as well as constructions that promote nutrient-transport rates, diffusion distances and vascularization [3].

Our recent studies have reported that lipoaspirate-derived adipose tissue (LAT) mixed with alginate and nanocellulose shows a high degree of 3D printability and *in vivo* survival (30 and 99 days), with the LAT bioink comprising putative ASCs (CD90^+^) [4,5]. The complexity of adipose tissue has become increasingly evident, with fat now considered a highly active endocrine and metabolic organ [6,7] that harbours depot-specific differences in proteome and secretome compositions [7–9]. There are limited studies characterizing the complete proteome of human white subcutaneous adipose tissue and the stromal vascular fraction (SVF) [9]. Specifically, the proteome of mechanically processed LAT has not previously been investigated.

Paracrine signalling by secretory proteins from both adipocytes and ASCs regulates cell renewal, differentiation and angiogenesis, as well as displays anti-inflammatory effects that potentially suppress graft resorption [7,10,11].

In this study, we explored the proteomic signature and cellular composition of LAT capable of potentially influencing *in vivo* survival, including the presence of angiogenic proteins and various growth factors (GFs), as well as ASCs, pericytes, endothelial progenitor cells (EPCs) and endothelial cells. In addition to determining the composition of microfractured LAT, we investigated the long-term *in vivo* survival and revascularization of 3D-bioprinted grafts in an animal model.

## Results

### Proteomic signature of mechanically processed LAT

Proteomic characterization of the LAT identified 6,067 different proteins, a selection of which is presented in Table 1. The proteome included several adipocyte-secreted proteins, including adipokines (visfatin, previously identified mainly in visceral adipose tissue [9]), complement factor D, retinol-binding protein 4, SPARC (osteonectin), adiponectin and retinoic acid receptor responder protein 2, which regulate various biological processes, including adipogenesis, adipocyte differentiation and angiogenesis [12,13]. Neither leptin nor omentin were detected, consistent with previous studies [8,14]. Additionally, we found several proteins involved in adipocyte differentiation, including adipocyte plasma-membrane-associated protein, adipocyte enhancer-binding protein 1, adipogenesis regulatory factor, angiopoietin-like protein (ANGPTL)8 and peroxisome proliferator-activated receptor (PPAR)γ, which is a master regulator of adipogenesis [15], a key regulator of adipocyte differentiation, and also involved in regulating epithelial cell differentiation, angiogenesis and cell growth [12]. Moreover, we also identified PPARα, which is a key regulator of lipid metabolism and is involved in epidermis development and wound healing [12]. Furthermore, several vitamin A-related proteins associated with adipocyte differentiation and survival were identified, including retinal dehydrogenases 1 and 2 (ALDH1A1/2), retinol-binding proteins 1 and 4, aldehyde dehydrogenase family 1 member A3 (aldehyde dehydrogenase 6), retinoic acid receptor RXR-β, retinol dehydrogenases 10, 11 and 14 and cellular retinoic acid-binding proteins 2 and 4 [16].

**Table 1.** Selected proteins identified in the LAT by nano-LC-MS/MS

Protein nameAdipokines	Functions^a^	Accession code^b^
Visfatin	Cell proliferation	P43490
Adiponectin	Hormone, cell growth, differentiation and angiogenesis	Q15848
Retinoic acid receptor responder protein 2	Regulates adipogenesis and adipocyte differentiation	Q99969
Complement factor D		P00746
Retinol-binding protein 4		P02753
SPARC	Cell growth	P09486
**Angiogenic proteins**		
TNF α- induced protein 2	Angiogenesis and differentiation	Q03169
Angiopoietin-1 (Ang-1)	Angiogenesis and cell survival	Q15389
Angiopoietin-2 (Ang-2)	Angiogenesis and differentiation	O15123
Angio-associated migratory cell protein	Angiogenesis and differentiation	Q13685
Angiopoietin-1 receptor	Angiogenesis and cell survival	Q15389
Angiopoietin-related protein 2	Angiogenesis	Q9UKU9
Angiopoietin-related protein 4	Angiogenesis and differentiation	Q9BY76
**Pericyte markers**		
Desmin	Muscle protein	P17661
Platelet-derived growth factor receptor β (PDGFR-β)	Cell proliferation and differentiation	P09619
Chondroitin sulphate proteoglycan 4 (NG2)	Angiogensis and cell proliferation	Q6UVK1
Aminopeptidase N (APN, CD13)	Angiogenesis and cell differentiation	P15144
Vimentin		P08670
Cell surface glycoprotein MUC18 (MCAM and CD146)	Angiogenesis	P43121
Endosialin (CD248)	Cell proliferation	Q9HCU0
Neurogenic locus notch homolog protein 3 (NOTCH3)	Angiogenesis and cell differentiation	Q9UM47
**Growth factors and associated proteins**		
Transforming growth factor β1 (TGF-β1)	Cell survival and proliferation	P01137
Fibroblast growth factor 1 (FGF-1)	Cell survival, angiogenesis and differentiation	P05230
Fibroblast growth factor 2 (FGF-2)	Cell survival, angiogenesis and differentiation	P09038
Epidermal growth factor-like protein 6	Differentiation	Q8IUX8
Epidermal growth factor-like protein 7	Angiogenesis	Q9UHF1
Hepatoma-derived growth factor (HDGF)	Cell proliferation	P51858
Hepatoma-derived growth factor-related protein 2 (HRP-2)	Cell growth	Q7Z4V5
Hepatoma-derived growth factor-related protein 3 (HRP-3)	Cell proliferation	Q9Y3E1
Myeloid-derived growth factor (MYDGF and IL-25)	Angiogenesis and cell proliferation	Q969H8
Stromal/stem cell-derived factor 1 (SDF-1)	Growth factor and chemokine activity	P48061
**Growth factor receptors**		
Fibroblast growth factor receptor 1 (FGFR-1)	Angiogenesis and differentiation	P11362
Vascular endothelial growth factor receptor 1 (VEGFR-1)	Angiogenesis, cell proliferation and survival	P17948
Vascular endothelial growth factor receptor 3 (VEGFR-3)	Lymphangiogenesis and angiogenesis	P35916
Insulin receptor	Cell proliferation	P06213
Insulin-like growth factor 1 receptor (IGF1R)	Cell proliferation	P08069
Epidermal growth factor receptor	Cell growth	P00533
Platelet-derived growth factor receptor β (PDGFR-β)	Cell proliferation and differentiation	P09619
Platelet-derived growth factor receptor α (PDGFR-α)	Cell proliferation and differentiation	P16234
TNF receptor superfamily member 1A	Angiogenesis and cell proliferation	P19438
TNF receptor superfamily member 5	Cell proliferation	P25942
**GF-binding proteins**		
Growth factor receptor-bound protein 2	Cell proliferation and differentiation	P62993
Insulin-like growth factor 2 mRNA-binding protein 2 (IGFBP-2)		Q9Y6M1
Insulin-like growth factor-binding protein 3 (IGFBP-3)	Cell growth, proliferation and differentation	P17936
Insulin-like growth factor-binding protein 5 (IGFBP-5)	Cell growth, proliferation and differentiation	P24593
Insulin-like growth factor-binding protein 6 (IGFBP-6)	Cell growth	P24592
Insulin-like growth factor-binding protein 7 (IGFBP-7)	Cell growth	Q16270
Acidic fibroblast growth factor intracellular-binding protein (FGF-1 intracellular-binding protein)		O43427
Latent-transforming growth factor beta-binding protein 1 (LTBP-1)	Structural role in ECM	Q14766
Latent-transforming growth factor beta-binding protein 2 (LTBP-2)	Structural role in ECM	Q14767
Latent-transforming growth factor beta-binding protein 3 (LTBP-3)	Structural role in ECM	Q9NS15
Latent-transforming growth factor beta-binding protein 4 (LTBP-4)	Cell growth anddifferentiation	Q8N2S1
TNF receptor-associated factor 2		Q12933
TNF receptor-associated factor 6	Osteoclast differentiation and smooth muscle proliferation	Q9Y4K3
TNF receptor type 1-associated DEATH domain protein		Q15628
**Extracellular matrix (ECM) proteins**		
Collagen type IV	Basement membrane associated	P02462 and P08572
Laminin α 2, 3, 4 and 5	Basement membrane associated	P24043, Q16787, Q16363 and O15230
Laminin β 1, 2 and 3	Basement membrane associated	P07942, P55268 and Q13751
Lamnin γ 1	Basement membrane associated	P11047
Agrin	Basement membrane associated	O00468
Perlecane (Basement membrane-specific heparan sulphate proteoglycan core protein)	Basement membrane associated and angiogenesis	P98160
Fibronectin	Basement membrane associated	P02751
Nidogen-1	Basement membrane associated	P14543
Nidogen-2	Basement membrane associated	Q14112

^a^According to the UniProtKB database. ^b^ Protein accession code from Swiss-Prot database

In addition to adipokines, the proteome included the ASC secretome comprising various GFs and angiogenic proteins (Table 1). We identified several proteins associated with the ASC secretome, including transforming GF-β1 (TGF-β1), although prostaglandin E2, granulocyte-macrophage colony-stimulating factor and interleukin (IL)-6, −7, −8, or −11 were not identified in the analysis. Additionally, we identified various angiogenic proteins secreted by ASCs, including fibroblast GF (FGF)-2, angiopoietin (ANG)-1 and −2 and vascular endothelial GF receptor (VEGFR)-1 and −3, although no VEGF, hepatocyte GF or insulin-like GF (IGF)-1 was found.

Moreover, we identified pericyte markers involved in endothelial and smooth muscle cell differentiation and angiogenesis, including desmin, platelet-derived GF receptor (PDGFR)-β [17], chondroitin sulphate proteoglycan 4 (NG2), aminopeptidase N (AP-N; CD13), actins and neurogenic locus notch homolog protein 3 (NOTCH3) [18,19]. Additional angiogenic proteins identified included angio-associated migratory cell protein (AAMP), tumour necrosis factor (TNF)-α-inducible protein 2, ANG-1 and −2, ANGPTL2 and ANGPTL 4, stromal-interaction molecule 1 [20], TGF-β-induced protein ig-h3 and angiomotin-like protein (AMOTL)1 and AMOTL2.

Several novel GFs involved in cell growth, survival, differentiation and angiogenesis were identified during proteomic analysis. These included paracrine-acting secretory proteins, such as FGF-1 and −2, hepatoma-derived GF (HDGF), HDGF-related proteins 2 and 3, myeloid-derived GF, stromal/stem cell-derived factor-1 and epidermal GF-like proteins 6 and 7. We did not find PDGF-D, which was previously identified as a novel protein of the adipocyte secretome [13]. The proteome also included numerous GF-binding proteins involved in GF-receptor signalling pathways, including IGF-binding protein-2/mRNA-binding protein 2 (IGFBP-2/IMP-2) and IGFBP-3, −5, −6 and −7. Furthermore, we identified novel proteins, including acidic FGF intracellular-binding protein and various TGF-β1-binding proteins, such as latent-TGF-β1-binding protein (LTBP)-1, −2, −3 and −4.

Additionally, we identified several GF receptors and associated binding proteins (Table 1). In addition to VEGFR-1, we identified VEGFR-3, FGF receptor-1, insulin receptor, IGF-1 receptor, epidermal GF receptor (EGFR), PDGFR-β and -α and proteins involved in the TNF-mediated signalling pathway, including TNF receptor superfamily members 1A and 5 and TNF receptor-associated factors 2 and 6.

Furthermore, we identified components of developmental morphogenic signalling pathways (i.e. Wnt, TGF-β and NOTCH) possibly involved in auto-paracrine regulation of adipose differentiation and angiogenesis [13]. Proteins involved in the Wnt signalling pathway included Wnt-11, protein Wnt-less homolog, secreted frizzled-related protein 1 and AMOTL1 and AMOTL2. In addition to TGF-β, other identified proteins involved in this signalling pathway included TGF-β1-induced transcript 1 protein (also involved in regulation of Wnt signalling), TGF-β- receptor-associated protein 1, LTBP, endoglin/CD105 and mothers against decapentaplegic homolog (SMAD)2/3/4/5. Additionally, identified proteins involved in NOTCH signalling included NOTCH2/3/4, EGFR (also involved in regulation of Wnt signalling) and EGF-like protein 7.

Interestingly, we found no glycosyltransferases essential for biosynthesis of histo-blood group ABO antigens. Therefore, neither α-1,2-fucosyltransferases (responsible for the H-antigen) nor α-1-3-acetylgalactosaminyltransferas (responsible for the A-antigen) or galactosyltransferases (responsible for the B-antigen) were present [21]. However, we identified both human leukocyte antigen classes I and II, indicating the presence of antigen-presenting cells, such as macrophages and consistent with histological findings described later.

We also identified various structural components of the extracellular matrix, such as fibronectin, nidogen (I, II), collagens (I, III, IV, V, VI, VIII, XII, XIV, XV, XVI and XVIII) and fibrillin-1, which plays structural and regulatory roles in connective tissues and is significant to tissue haemostasis via interactions with different GFs and LTBPs involved in TGF-β sequestration and activation [22]. Surprisingly, we found cytokeratin (CK)-8 and −18, previously found only in omental/visceral adipose tissue and not in subcutaneous adipose tissues [8,23], as well as ezrin and vesicle amine transport-1, previously identified only in single studies of white adipose tissues of subcutaneous origin [8]. Additionally, we identified CK-1, −1b, −2, −4, −5, −6A, −6B, −6 C, −9, −10, −14, −16, −17, −23, −27, −71, −72, −78 and −80.

Other previously identified secretory proteins identified in this study included pigment epithelium-derived factor and macrophage migration-inhibitory factor [13]. Interestingly, we identified plasminogen activator inhibitor (PAI)-2, which is involved in fibrinolysis and wound healing, but not PAI-1, which was previously identified in white adipose tissue [7,24].

### Cellular content of mechanically processed LAT

**Figure 1.**
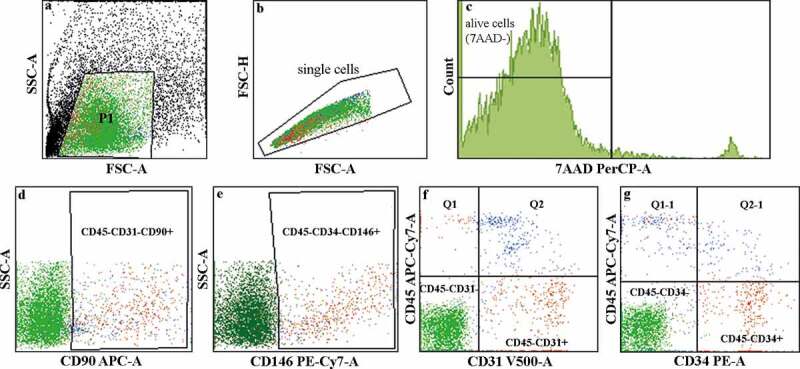
**Flow cytometry analysis of the cellular content of LAT**. (a–c) Illustration describing the gating process. The viability assay using 7AAD staining showed 96.6% cell viability in the LAT. The live-cell populations (7AAD^−^) identified in the LAT used in the *in vivo* study comprised (d) 1% ASCs (CD45^−^CD31^−^CD90^+^), (e) 1% pericytes (CD34^−^ CD45^−^CD146^+^), (f) 3.2% EPCs (CD45^−^CD34^+^) and (g) 3.6% endothelial cells (CD45^−^CD31^+^).

Processing of lipoaspirate with the Lipogems system resulted in 12 mL to 15 mL of LAT, with the total cell count ranging from 0.8 × 10^6^ to 4.5 × 10^6^ cells in the infranatant following collagenase treatment and red blood cell lysis (exclusion of adipocytes and erythrocytes). Analysis of the LAT (used in the *in vivo* study) using the NucleoCounter and 7-aminoactinomycin D (7AAD)^+^-exclusion methods indicated 96.6% viability and flow cytometry analysis revealed 1% ASCs (CD31^−^CD45^−^CD90^+^), 1% pericytes (CD34^−^CD45^−^CD146^+^), 3.2% EPCs (CD45^−^CD34^+^) and 3.6% endothelial cells (CD45^−^CD31^+^) (Figure 1). Furthermore, additional flow cytometry analysis of LAT from two different donors (3 and 4) identified 11.4% and 5.9% ASCs, 4.4% and 0.5% pericytes, 11.8% and 43.9% EPCs and 5.6% and 46.5% endothelial cells, respectively (Fig. S1).

### 3D bioprinting

The LAT showed good printability when combined with alginate and nanocellulose and the bioink containing adipose tissue allowed successful 3D bioprinting of both gridded and solid constructs (Fig. S2).

### Macroscopic, histologic and immunohistochemical evaluation

**Figure 2.**
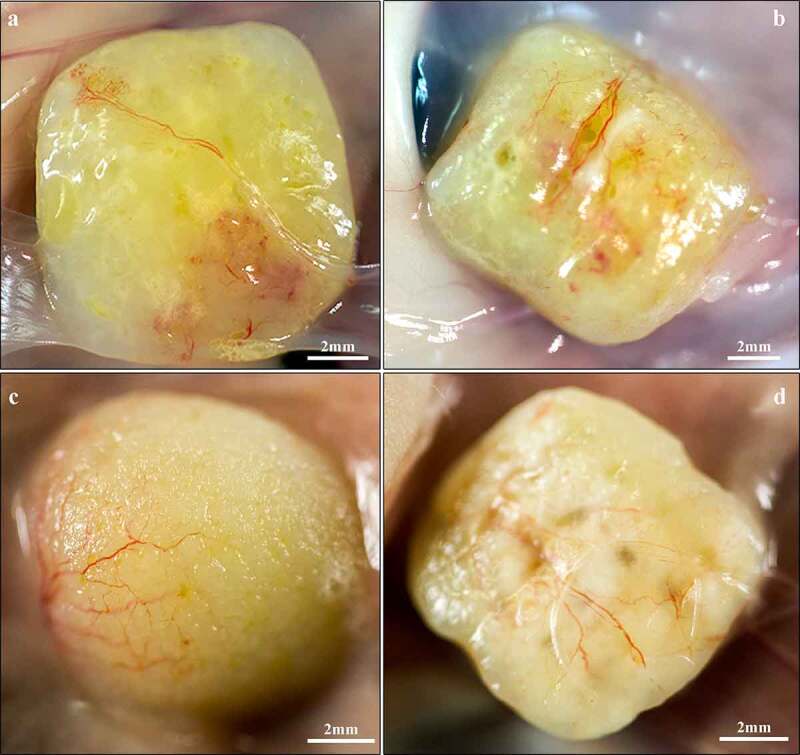
**Macroscopic images of explanted 3D-bioprinted LAT after 30** (a,b) **and 150** (c,d) **days *in vivo***. (a,c) Solid and (b,d) gridded constructs appeared intact along with a yellow fat-tissue-like appearance and preserved dimensions. The grid formations remained visible after 150 days *in vivo* and superficial blood vessels were present in the surrounding capsule.

**Figure 3.**
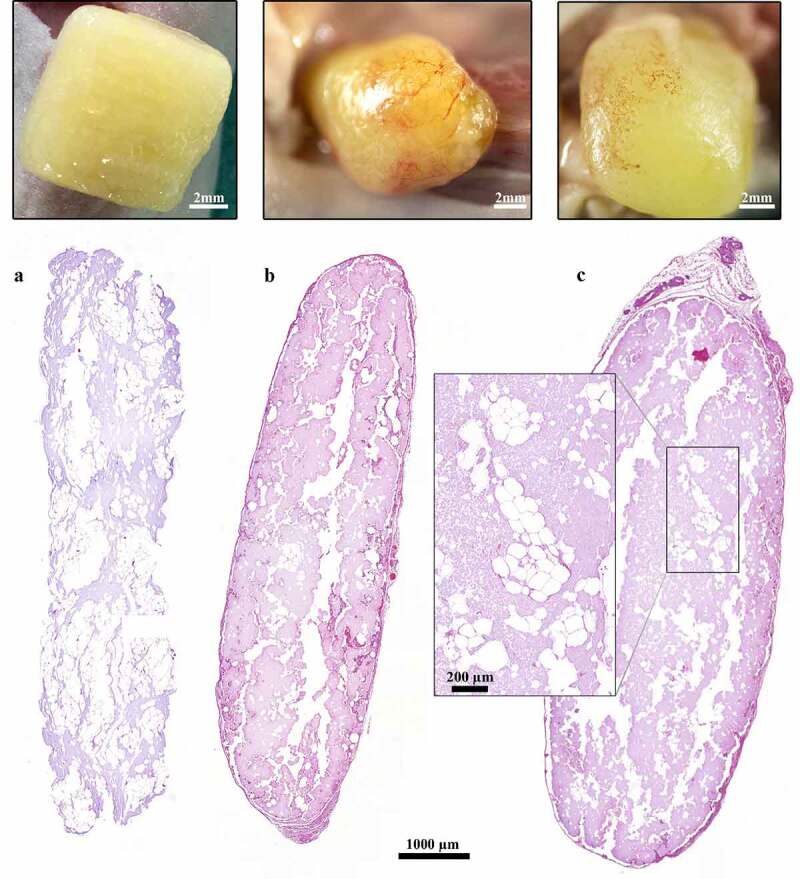
**Histological and macroscopic images of 3D-bioprinted LAT after printing and 30 and 150 days *in vivo***. (a–c) The dimensions of the constructs were preserved and adipocytes survived in the grafts for (b) 30 and 150 days *in vivo*; however, the presence of adipocytes declined from days (a) 0 to (c) 150. The images were cropped and linearly adjusted for exposure and contrast.

The 3D-bioprinted LAT survived *in vivo* for 150 days along with preserved size, shape and pore geometry (Figure 2). The presence of adipocytes declined between days 30 and 150 (Figure 3) and in some printed constructs, the tissue was almost totally resorbed and replaced by fibrotic tissue (Fig. S3). One animal (gridded; day-30 group) died of procedure-related intraperitoneal bleeding.

The 3D-bioprinted LAT grafts contained intact vascular structures and mature adipocytes before engraftment and after explantation at days 30 and 150 (Figures 2 and 3).

**Figure 4.**
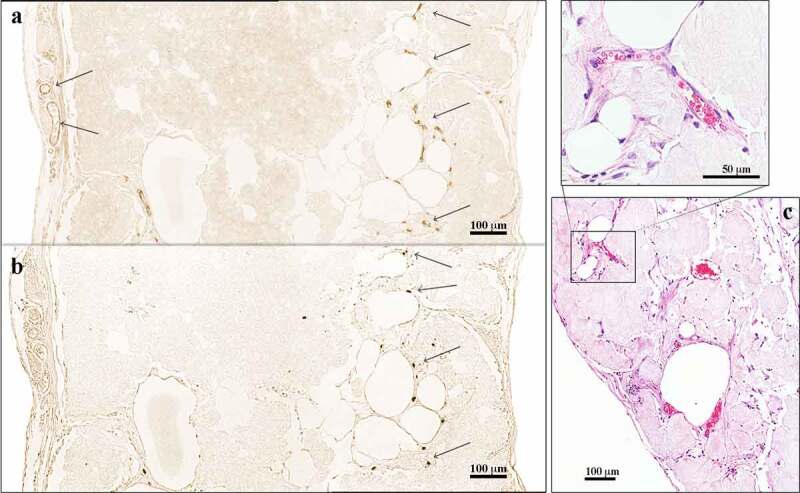
**Immunohistochemical analysis of 3D-bioprinted LAT explanted after 30 days *in vivo***. (a) The endothelial marker CD31 was used to visualize blood vessels apparent in the capsule and the centre of the construct (arrows). (b) Consecutive sections were stained with the anti-human specific marker Ku80. The blood vessels in the capsule were of mouse origin (Ku80^−^), whereas the vascular structures present in the centre of the constructs were human (Ku80^+^). The images were cropped and linearly adjusted for exposure and contrast. (c) Blood vessels with erythrocytes in the lumen were observed in 3D-bioprinted LAT constructs explanted after 150 days *in vivo*, indicating functioning blood vessels connected to mouse systemic circulation. The images were cropped and linearly adjusted for exposure and contrast.

After 30 days *in vivo*, novel blood vessels were macroscopically visible on the graft surfaces and showed signs of angiogenesis into the graft, as well as vascularization in the centre of the tissue (Figures 2 and 4). Additionally, we observed blood vessels in the capsule, as well as in the centre of the construct (Figure 4a, b), with some of these vessels harbouring erythrocytes in the lumen (Figure 4c). These findings indicated the presence of functional blood vessels connected to the systemic circulation of the mice.

A majority of blood vessels stained with the anti-CD31 antibody (endothelial cell marker) were of mouse origin and consequently negative for Ku80 (human-specific biomarker [25]) in consecutive slides. Interestingly, some vascular structures mainly located in the central areas of the constructs showed Ku80^+^ staining (Figure 4). Furthermore, macroscopic and histologic evaluations showed a transformation from adipose tissue to more fibrotic tissue over time (Figures 2 and 3); however, viable and intact adipocytes remained present at day 150 (Figure 3).

### Discussion

This study confirmed the complex composition of LAT as not only a volumizing filler but also a source of multipotent stem cells and multiple biologically active elements involved in paracrine signalling to regulate cell growth, differentiation and angiogenesis, as well as anti-inflammatory properties. The results demonstrated that LAT comprises cell types and a proteomic composition that could potentially positively influence autologous fat-graft survival.

Recent perception of adipose tissue as merely a passive source of energy and insulation has been challenged and revised. Adipose tissue is now defined as an endocrine organ with extensive networks of auto-paracrine signalling pathways [6,7], as well as a rich variety of cell types, including ASCs and EPCs [26,27]. The reservoir of multipotent adult mesenchymal stem cells found in adipose tissue (i.e. ASCs) exhibits extensive self-renewal, trophic paracrine and multi-linage differentiation capacities [28]. Indeed, ASCs represent the highest percentage of adult stem cells found in the human body, far exceeding that of classical bone marrow-derived mesenchymal stem cells [29]. Additionally, adipose tissue harbours resident EPCs important for neovascularization and angiogenesis [30], as circulating EPCs can potentially be home to sites of neovascularization and differentiate into endothelial cells [31]. In the present study, flow cytometric analysis of microfractured and non-enzymatically produced LAT confirmed the presence of ASCs (CD31^−^CD45^−^CD90^+^), pericytes (CD34^−^CD45^−^CD146^+^), EPCs (CD45^−^CD34^+^) and endothelial cells (CD45^−^CD31^+^) as tissue-resident cell types involved in graft survival, cell growth and angiogenesis [27,28]. Because the cell composition and concentration in lipoaspirate can be influenced by the donor site [32], we performed flow cytometric analysis of LAT harvested from the same donor sites in three females in order to determine the cellular content. The results confirmed the interpersonal variance in the cellular composition and the presence of ASCs and EPCs.

Autologous fat grafting with adipose tissue prepared from human lipoaspirate via the Coleman technique [1] is a well-established procedure for correction of soft-tissue defects in reconstructive surgery. However, long-term graft survival and volume retention vary and are often insufficient, subsequently requiring multiple corrections [2]. Various methods for optimizing the lipoaspirate, such as enrichment with ASCs or enzymatically or mechanically (e.g. the Lipogems system) isolating the SVF, are currently applied clinically [33–37]. Zhang et al. [38] showed that SVF grafts exhibit a long-term volume-retention rate superior to conventional Coleman fat, stimulate angiogenesis and induce host-cell-mediated adipogenesis. Consistency with our previous studies [4,5], the present study showed that LAT-containing constructs retained their size and shape for 150 days, which is equivalent to ~33% of the expected lifespan of the animal model used [39]. Macroscopic evaluation findings at 150-days post-engraftment showed adipose tissue with consistent volume and persistent pore formations. Additionally, the biomaterial allowed spatial separation of cells and structures in the bioprinted construct that contributed to the successful preservation of size and shape (i.e. the spatial separation promoted survival due to the favourable diffusion of oxygen and nutrients). These results should be compared with conventional human autologous fat grafts with reabsorption rates ranging from 50% to 70% after 1 year [40]. However, qualitative histologic evaluation showed a tendency of decreasing presence of adipocytes between days 30 and 150, which could be a consequence of ageing and fibrotic transformation and requires further quantitative evaluation. Furthermore, a limitation of the study was the lack of a control group undergoing conventional autologous fat grafting (i.e. injected and not 3D-bioprinted LAT), with establishment of such a control hindered by the loose skin of the nude mice and their sparse subcutaneous layer. Instead, we used 3D-bioprinted LAT from day 0 constructs (*n* = 12) as a baseline for the *in vivo* study.

In agreement with the findings of our previous study [4,5], in the present study, we observed functional blood vessels containing erythrocytes inside of grafts, with an in-growth of blood vessels of murine origin (i.e. Ku80^−^ staining) found in the surrounding fibrotic capsule. Moreover, we found neovascularization of human origin in the central parts of the grafts according to Ku80^+^ staining [25]. This suggested preservation of pre-existing blood vessels in the LAT and their survival during the 3D-bioprinting process to facilitate angiogenesis *in vivo*. Furthermore, angiogenesis could potentially be stimulated by resident human ASCs through paracrine action or their capacity to differentiate into endothelial cells [28].

Given the flow cytometric and histological findings of the 3D-bioprinted adipose tissue showing signs of neovascularization following engraftment and long-term survival, we analysed the LAT to determine the content of mainly secretory proteins involved in cell growth, differentiation and angiogenesis. The proteome comprised 6,067 different proteins, including several adipokines and members of the ASC secretome that included various GFs and angiogenic proteins. Furthermore, we identified several pericyte markers, including PDGFR-β [17], NG2, AP-N and desmin [18]. Analogous to ASCs, pericytes are a cell type of special interest due to their critical roles in angiogenesis, wound healing, inflammation and fibrosis [18,41]. Pericytes and endothelial cells collectively create and maintain the basement membrane of the vessel wall, which is crucial for vascular formation and maturation [42]. Proteomic analysis identified essential constituents of the basement membrane, including laminins, type IV collagens, perlecan, agrin and nidogens [43,44]. Additionally, the structural significance of laminins in the basement membrane involves their cell-regulatory functions and roles in various cellular processes, such as differentiation and phenotype maintenance [45]. Additionally, laminins are established stem cell-culture substrates that facilitate adhesion, expansion and survival [46,47], suggesting their potential positive influence on resident ASCs.

Furthermore, proteomic analysis identified several proteins involved in retinoid metabolism associated with adipocyte differentiation and survival, as well as components of developmental morphogenic signalling pathways (i.e. NOTCH, Wnt and TGF-β, of which the latter two are known to coordinate adipose differentiation by auto-paracrine actions) [13]. We identified several LTBP(1–4) and SMAD(2–5) proteins that also regulate proliferation and differentiation of human mesenchymal stem cells (e.g. ASCs) through the TGF-β signalling pathway [48]. Additionally, the NOTCH signalling pathway is essential for angiogenesis [49] and ASC-driven vascularization, with NOTCH2 specifically identified and reported to influence the capacity of ASCs to promote endothelial networks and a pericytic phenotype [50].

Moreover, we identified various proteins with developmental molecular functions involved in angiogenesis and differentiation [i.e. TNF-α-induced protein 2, FGF-1 and −2, ANG-1 and −2, VEGFR-1, AAMP, angiopoietin-related protein 4, PDGFR-β, PDGFR-α and AP-N (CD13)]. Additionally, several novel proteins were identified in LAT, including GFs and GF-binding proteins (visfatin, ezrin, VAT-1 and CK-8 and −18, both of which have been mainly described in visceral adipose tissue), and not previously described in subcutaneous tissue [8,23].

Few previous studies have characterized the entire proteome of human white subcutaneous adipose tissue and the SVF [9,51,52], partly due to the technical challenges related to high lipid content. Notably, the present study represents the first proteomic analysis using LC-MS/MS of mechanically processed LAT, in general, or a Lipogems product, in particular. The detailed information provided confirmed that LAT grafts are active and complex endocrine tissue and a basis for understanding the full capacity of future 3D bioprinting with adipose tissue.

Mechanically isolated SVF lacks the regulatory issues associated with the enzymatically produced equivalent and is already in clinical use [33–37]. Similarly, LAT isolated using the Lipogems® system used in this study is currently being evaluated by various ongoing clinical trials and applications [34]. Therefore, this study demonstrated a LAT-isolation method currently in clinical use and without major legislative obstacles, revealing that LAT exhibited favourable cellular and proteomic contents that can potentially promote survival and be 3D bioprinted into custom-made grafts that show long-term retention of size and shape.

### Conclusions

In conclusion, we described how mechanically processed human fat can be bioprinted into a customized 3D size and shape ultimately preserved following *in vivo* engraftment. The cellular composition and proteomic profile of the LAT in combination with the favourable properties of the biomaterial can promote angiogenesis and fat-graft survival.

## Materials and methods

### LAT preparation

The bioink was prepared according to a previously described method [4]. Briefly, waste human lipoaspirate was harvested from the abdomen and flanks of four healthy donors by conventional water-jet-assisted techniques and Klein’s standard tumescent solution following approval from the Regional Ethics Committee of Gothenburg (Dnr 624–16) and after receipt of written informed consent. The study was performed in accordance with institutional, national and European guidelines and regulations including the Helsinki Declaration of 1975. The lipoaspirate was mechanically processed with a Lipogems kit according to the manufacturer’s instructions (Lipogems International SpA, Milan, Italy) [4,53,54]. The proteomic signature and cellular content of the obtained LAT were analysed by proteomics (donor 1) and flow cytometry (donors 2–4). Furthermore, LAT (donor 2) was 3D bioprinted and subsequently used for *in vivo* studies.

### Protein digestion, peptide labelling and fractionation

The LAT sample was homogenized in a total volume of 500 µL of lysis buffer [final concentrations: 2% sodium dodecyl sulphate and 25 mM triethylammonium bicarbonate (TEAB)] with 1.4-mm ceramic spheres (FastPrep matrix D) using a FastPrep-24 instrument (MP Biomedicals, Irvina, CA, USA). The supernatant was collected after centrifugation at 13,000 for 10 min, after which the beads were washed with the lysis buffer and spun at 13,000 rpm for 10 min. The washing buffer was then combined with the supernatant. The protein concentration was determined using a Pierce BCA protein assay (Thermo Fisher Scientific, Waltham, MA, USA) and a Benchmark Plus microplate reader (Bio-Rad, Hercules, CA, USA) with bovine serum albumin (BSA) used as a standard. An aliquot of 30 µg of total protein was digested with trypsin using the filter-aided sample preparation method [55]. Briefly, protein samples were reduced with 100 mM dithiothreitol at 60°C for 30 min, transferred to 30-kDa molecular-weight cut-off Nanosep centrifugal filters (Pall Life Sciences, Portsmouth, UK), repeatedly washed with 8 M urea solution and alkylated with 10 mM methyl methanethiosulfonate in digestion buffer [50 mM TEAB and 1% sodium deoxycholate (SDC)]. The filters were spun at 10,000 rpm at room temperature for 3–10 min in each step. Digestion was performed by the addition of Pierce MS-grade trypsin (0.20 µg; Thermo Fisher Scientific) in digestion buffer at a 1:100 (v/w) ratio relative to the protein content and incubated overnight at 37°C, followed by the addition of more trypsin and incubation for another 2 h. Peptides were collected by centrifugation at 12,000 rpm at room temperature for 20 min. SDC was removed by acidification with 10% trifluoroacetic acid and the sample was fractionated into 20 fractions using basic reversed-phase liquid chromatography (LC) using a Dionex Ultimate 3000 ultra-performance LC system (Thermo Fisher Scientific). Peptide separations were performed using a reversed-phase XBridge BEH C18 column (3.5 μm, 3.0 × 150 mm; Waters Corporation, Milford, MA, USA) and a linear gradient from 3% to 40% of solvent B [90% acetonitrile and 10 mM ammonium formate (10%) (pH 10.0)] and consequently 97% to 60% of solvent A [10 mM ammonium formate buffer (pH 10.00)] for 17 min, followed by an increase to 100% solvent B for 5 min. The fractions were then dried and reconstituted in 3% acetonitrile and 0.2% formic acid for nano-LC-tandem mass spectrometry (LC-MS/MS) analysis.

### Proteomic analysis

Descriptive characterization of the proteomic composition of the LAT was performed using two-dimensional LC-MS/MS. The fractions were analysed using a QExactive HF mass spectrometer interfaced with an Easy-nLC1200 LC system (Thermo Fisher Scientific). Peptides were trapped on an Acclaim Pepmap 100 C18 trap column (100 μm × 2 cm; particle size, 5 μm; Thermo Fisher Scientific) and separated on an in-house-packed analytical column (75 μm × 300 mm; particle size, 3 μm; Reprosil-Pur C18; Dr. Maisch HPLC GmbH, Ammerbuch, Germany) using a linear gradient from 7% to 45% of solvent B [80% acetonitrile in 0.2% formic acid] and consequently 93% to 55% of solvent A [0.2% formic acid] for 75 min, followed by an increase to 100% solvent B for 5 min at a flow rate of 300 nL/min. The instrument was operated in data-dependent mode, with precursor ion mass spectra acquired at a resolution of 60,000. The 10 most intense ions were isolated in a 1.2-Da isolation window and fragmented using a higher-energy collisional dissociation setting of 28. MS/MS spectra were recorded at a resolution of 30,000, charge states from 2 to 4 were selected for fragmentation and dynamic exclusion was set to 20 s. Data analysis was performed using Proteome Discoverer (v.1.4; Thermo Fisher Scientific) against the Human Swissprot Database (v.Nov 2017; Swiss Institute of Bioinformatics, Lausanne, Switzerland). Mascot 2.5 (Matrix Science, Boston, MA, USA) was used as a search engine and using a precursor mass tolerance of 5 ppm and a fragment mass tolerance of 200 mmu. Tryptic peptides were accepted with one missed cleavage and variable modification of methionine oxidation and fixed cysteine alkylation was selected. The detected peptide threshold in the software was set to a 1% false discovery rate by searching against a reversed database and identified proteins were grouped by sharing the same sequences to minimize redundancy. Selected proteins were further classified according to the UniProtKB database [12].

### Flow cytometry analysis

To evaluate the LAT cellular composition, we performed multicolour flow cytometry analysis on LAT from three different donors. Adipose tissue was treated with 0.075% collagenase 1A (Gibco, Gaithersburg, MD, USA) at 37°C for 30 min, after which the infranatant was collected and collagenase activity was stopped with Dulbecco’s modified Eagle medium (Gibco) supplemented with 10% foetal bovine serum (Hyclone Laboratories, Logan, UT, USA). The infranatant was centrifuged and cells were washed with phosphate-buffered saline (PBS), followed by resuspension in PBS supplemented with 0.5% BSA before fluorescence-activated cell sorting (FACS) analysis. The total cell count was evaluated using the Countess automated cell counter (Thermo Fisher Science). Following collagenase treatment, the LAT was phenotypically characterized for antigens associated with ASCs (CD31^−^CD45^−^CD90^+^ [26]), pericytes (CD34^−^CD45^−^CD146^+^ [56]), EPCs (CD45^−^CD34^+^ [27,30,57]) and endothelial cells (CD45^−^CD31^+^) by multicolour flow cytometry. Briefly, 5 mL of the processed LAT was washed with PBS and centrifuged for 5 min at 250 *g* (centrifuge 5702 R; Eppendorf AG, Hamburg, Germany). The cell pellet and liquid underlying the supernatant and containing mature adipocytes were aspirated and collected, the remaining supernatant was washed three times and the cell suspensions were pooled. Cell count and viability were determined using a NucleoCounter NC-200 system (ChemoMetech, Lillerød, Denmark). A portion of the cell suspension (200 µL) containing ~2 × 10^5^ cells was distributed in separate polystyrene tubes (5 mL; 12 × 75 mm; Sarstedt AG & Co., Nümbrecht, Germany). Conjugated monoclonal antibodies from BD Biosciences (Franklin Lakes, NJ, USA) [FITC-anti CD105 (cat. 561,443, clone 266), PE-anti CD34 (cat. 345,802, clone 8G12), anti-CD146 (cat. 562,135, clone P1H12), anti-CD90 (cat. 559,869, clone 5E10), anti-CD45 (cat. 348,815, clone 2D1), anti-CD44 (cat. 561,292, clone G4426) and anti-CD31(cat. 562,454, clone WM59)] were added to the tubes, the samples were mixed and the cells were incubated at room temperature in the dark for 15 min. The cells were then washed with 2 mL PBS supplemented with 0.5% BSA once and centrifuged at 300 *g* for 5 min. The pellets were resuspended with 200 µL PBS supplemented with 0.5% BSA and then passed through a cell strainer to separate non-dissociated clumps (FALCON; 35 µm; Thermo Fisher Scientific). We then added 20 µL of the cell-viability marker 7AAD (cat. 555,816; BD Biosciences) to the tubes prior to flow cytometry (FACSaria II; BD Biosciences). Duplicate samples were prepared and an unstained sample was used as a gating control. We acquired 20,000 events for each sample and the fluorescence signals were analysed using FACSDiva software (BD Biosciences).

### *Bioink preparation and 3D bioprinting for* in vivo *studies*

After preparation, the obtained LAT was gently mixed with 3% (w/v) alginate solution prepared using lyophilized sterile sodium alginate powder (Pronova SLG100; DuPont NovaMatrix, Sandvika,

Norway) and 2.5% (w/v) medical-grade tunicate nanocellulose dispersion in water (TUNICELL ETC; Ocean TuniCell AS, Blomsterdalen, Norway) at a volumetric ratio of 45:15:40 (LAT:alginate:nanocellulose solutions/dispersions), as previously described [4]. The bioink was 3D bioprinted with an INKREDIBLE extrusion 3D bioprinter (CELLINK, Gothenburg, Sweden) as solid or gridded constructs with 1.45-mm line spacing (10 × 10 × 3 mm, respectively). An 18 G conical nozzle was used for printing at a printing pressure ranging from 5 kPa to 7 kPa. Bioprinted constructs were cross-linked with 100 mM CaCl_2_ for 5 min, rinsed with Hank’s balanced salt solution and immediately implanted subcutaneously in BALB/c nude mice (*n* = 36). Twelve ungrafted constructs (6 gridded and 6 solid; referred to as day 0 samples) were subsequently used for baseline evaluation in histologic and immunohistochemical analyses.

### Animal model

The 3D-bioprinted LAT constructs were implanted subcutaneously in the neck region of 8-week-old BALB/c nude mice (Scanbur, Karlslunde, Denmark) and harvested at days 30 (*n* = 19; 10 gridded and 9 solid constructs) and 150 (*n* = 15; 7 gridded and 8 solid constructs) under general anaesthesia, as previously described [4,58]. Thereafter, the explanted grafts were histologically and immunohistochemically evaluated. No antibiotics were used. The study was approved by the Ethics Committee for animal experiments at Sahlgrenska University Hospital (Gothenburg University, Göteborg, Sweden; Dnr 119–2015, 36–2016). All animal experiments were performed in accordance with institutional, national, European and ARRIVE guidelines [59] and regulations at the core facility for experimental biomedicine at the University of Gothenburg.

### Histologic and Immunohistochemical evaluation

Histologic analysis with haematoxylin and eosin staining was performed to investigate the morphological characteristics of the constructs and alterations *in vivo* after 0, 30 and 150 days. Additionally, immunohistochemical analysis of the constructs was performed, as previously described [4], to characterize the presence of endothelial cells (CD31^+^) and consequent vascular structures, as well as distinguish human-from mouse-origin vasculature using the human-specific biomarker Ku80 [25], in consecutive slides. Briefly, the 3D-bioprinted LAT constructs were explanted at days 30 (*n* = 19) and 150 (*n* = 15), fixed in buffered 4% paraformaldehyde and dehydrated and embedded in paraffin. Subsequently, 5-µm sections were mounted on Superfrost Plus glass slides (Menzel microscope slides; Thermo Fisher Scientific) and microwave-treated for antigen retrieval. Slides were stained with haematoxylin (Histolab Products AB, Gothenburg, Sweden) and eosin (Merck Millipore, Billerica, MA, USA) reagents for histologic evaluation. Immunostaining was performed after citrate pretreatment using a rabbit monoclonal anti-CD31 antibody (1:150; ab134168, clone EP3095; Abcam, Cambridge, UK), rabbit monoclonal anti-Ku80 (1:800; cat. 2180, clone C48E7; Cell Signalling Technology, Danvers, MA, USA), Vectastain Elite ABC kit peroxidase (PK-6101; Vector Laboratories, Burlingame, CA, USA) and ImmPACT DAB substrate (horseradish peroxidase; SK-4104; Vector Laboratories). Corresponding anti-rabbit IgG isotype controls (1:150; ab172730; Abcam) and human liver tissue (Fig. S4) were used as negative and positive controls, respectively.

## Supplementary Material

Supplemental Material

## Data Availability

The authors confirm that the data supporting the findings of this study are available within the article and its supplementary materials.
