# Supplementary material for: Long-term in vivo survival of 3D-bioprinted human lipoaspirate-derived adipose tissue: proteomic signature and cellular content
Source: Adipocyte. 2021 Dec 27;11(1):34–46. doi: 10.1080/21623945.2021.2014179 (PMC8726626; doi:10.1080/21623945.2021.2014179)
Supplement: Supplemental Material [file KADI_A_2014179_SM5539.zip › supplementary/Supplementary material .docx]

**Supplementary material**

**Long-term *in vivo* survival of 3D-bioprinted human lipoaspirate-derived adipose tissue: proteomic signature and cellular content**

Karin Säljö, Peter Apelgren, Linnéa Stridh Orrhult, Susann Li, Matteo Amoroso, Paul Gatenholm and Lars Kölby

**Figure S1. Flow cytometry analysis of LAT prepared from lipoaspirate harvested from the abdomen and flank of two healthy female donors. (a)** The LAT comprised of 11.4% ASCs (CD45^−^CD31^−^CD90^+^), 4.4% pericytes (CD34^−^ CD45^−^CD146^+^), 11.8% EPCs (CD45^−^CD34^+^), and 5.6% endothelial cells (CD45^−^CD31^+^). **(b)** The LAT comprised of 5.9% ASCs (CD45^−^CD31^−^CD90^+^), 0.5% pericytes (CD34^−^ CD45^−^CD146^+^), 43.9% EPCs (CD45^−^CD34^+^), and 46.5% endothelial cells (CD45^−^CD31^+^).


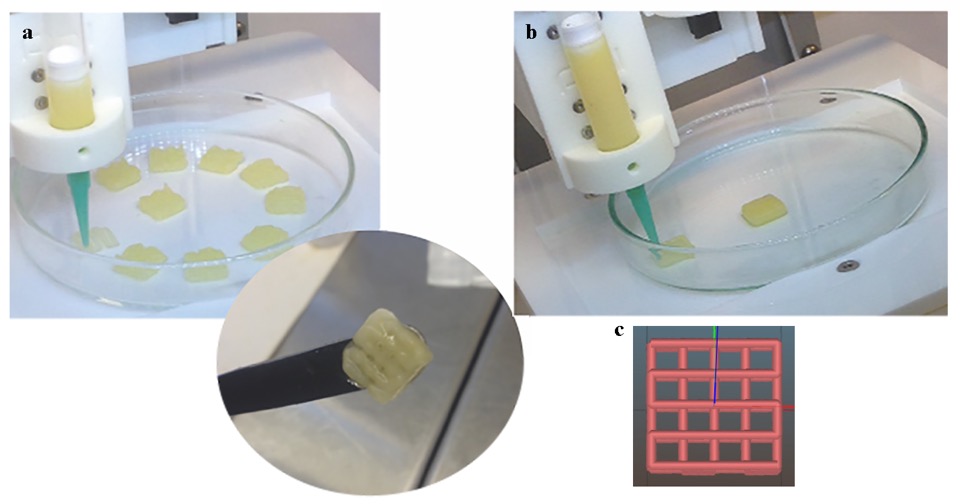


**Figure S2. 3D bioprinting of LAT onto constructs.** LAT bioprinting onto **(a)** gridded and **(b)** solid constructs. **(c)** The gridded geometry of the constructs as depicted by G-code. The bioink showed good viscosity, and the grid formations were distinct and similar in size and dimension.


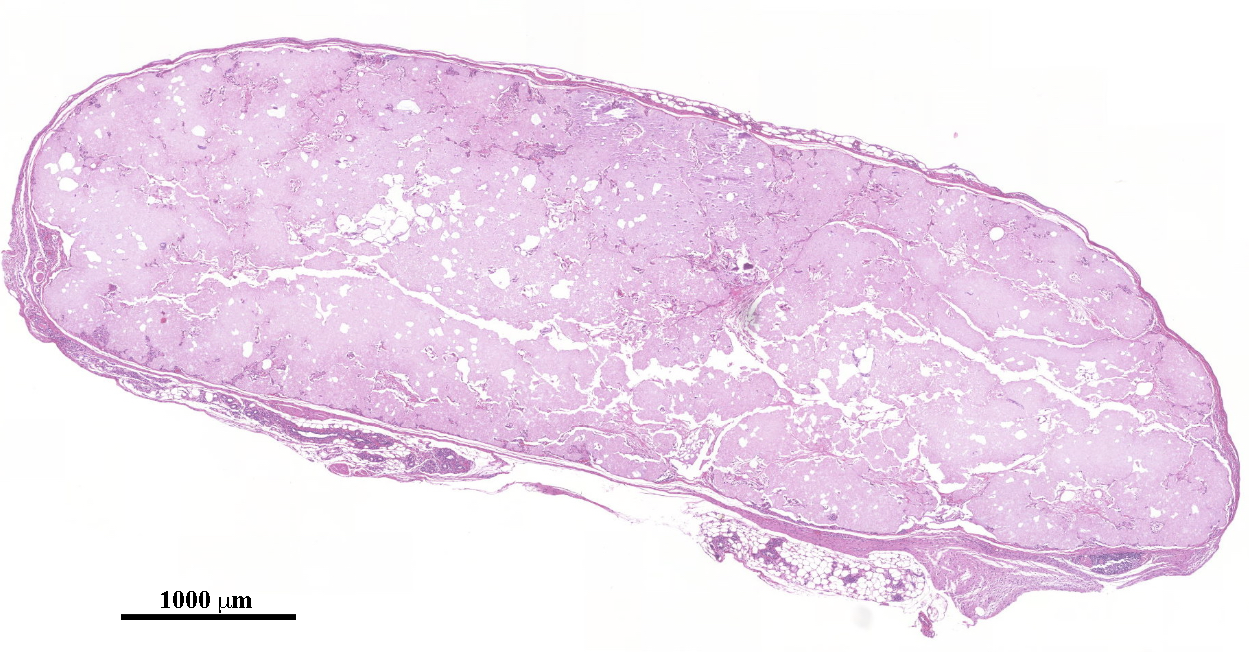


**Figure S3. A 3D-bioprinted LAT graft after 150 days *in vivo***. The graft showed signs of almost total resorption of adipocytes and fibrotic transformation, as well as size preservation.

**
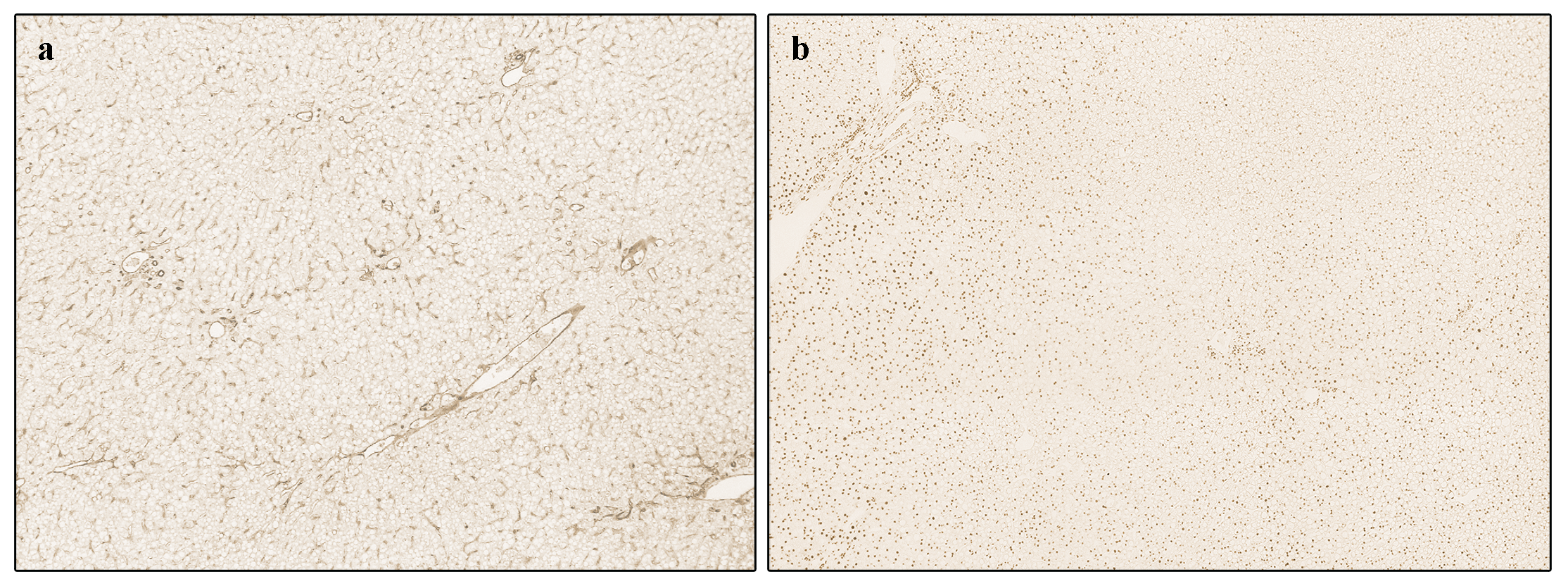
**

**Figure S4. Immunohistochemical staining of human liver tissue.** Human liver tissue sample stained with (**a**) rabbit monoclonal anti-CD31 antibody (positive control) and (**b**) anti-Ku-80.
